# Chemical Characterization, Antibacterial Activity, and Embryo Acute Toxicity of *Rhus coriaria* L. Genotype from Sicily (Italy)

**DOI:** 10.3390/foods11040538

**Published:** 2022-02-14

**Authors:** Giovanna Lo Vecchio, Nicola Cicero, Vincenzo Nava, Antonio Macrì, Claudio Gervasi, Fabiano Capparucci, Marzia Sciortino, Giuseppe Avellone, Qada Benameur, Antonello Santini, Teresa Gervasi

**Affiliations:** 1 Department of Biomedical and Dental Sciences and Morphofunctional Imaging, University of Messina, 98125 Messina, Italy; giolovecchio@unime.it (G.L.V.); vincenzo.nava@unime.it (V.N.); antonio.macri@unime.it (A.M.); teresa.gervasi@unime.it (T.G.); 2 Department of Chemical, Biological, Pharmaceutical and Environmental Sciences, University of Messina, 98166 Messina, Italy; claudio.gervasi@unime.it (C.G.); fabiano.capparucci@unime.it (F.C.); 3 Department of Biological, Chemical and Pharmaceutical Sciences and Technologies, University of Palermo, 90123 Palermo, Italy; marziasciortino@gmail.com (M.S.); beppe.avellone@unipa.it (G.A.); 4 Nursing Department, Faculty of Nature and Life Sciences, University of Mostaganem, Mostaganem 27000, Algeria; qada.benameur@univ-mosta.dz; 5 Department of Pharmacy, University of Napoli Federico II, Via D. Montesano 49, 80131 Napoli, Italy

**Keywords:** *Rhus coriaria*, sumac, polyphenols, antibacterial activity

## Abstract

This study reports a full characterization of the Sicilian sumac, *Rhus coriaria* L. This fruit represents a potential source of fiber (33.21 ± 1.02%) and unsaturated fatty acids, being the contents of linoleic and α-linolenic acids, 30.82 ± 1.21% and 1.85 ± 0.07%, respectively. In addition, the content of phenolic and total anthocyanin was 71.69 ± 1.23 mg/g as gallic acid equivalents, and 6.71 ± 0.12 mg/g as cyanidin-3-O-glucoside equivalents, respectively. The high content in mineral elements, consisting mainly of potassium, calcium, magnesium, and phosphorus, followed by aluminum, iron, sodium, boron, and zinc, was detected by inductively coupled plasma mass spectrometry (ICP-MS). Moreover, its antimicrobial activity was evaluated against multidrug resistant (MDR) microorganisms, represented by *Escherichia coli* and *Klebsiella pneumoniae* strains isolated from poultry. The activity of seven different sumac fruit extracts obtained using the following solvents—ethanol (SE), methanol (SM), acetone (SA), ethanol and water (SEW), methanol and water (SMW), acetone and water (SAW), water (SW)—was evaluated. The polyphenol profile of SM extract, which showed better activity, was analyzed by ultra-high performance liquid chromatography coupled with mass spectrometry (UHPLC-MS). The major component identified was gallic acid, followed by quercetin, methyl digallate, pentagalloyl-hexoside, and kaempferol 3-O-glucoside. The non-toxicity of Sicilian *R. coriaria* was confirmed by testing the effect of the same extract on zebrafish embryos.

## 1. Introduction

*Rhus coriaria* L., generally known as sumac, is a typical plant native of a large area spreading from the Canary Islands over the Mediterranean coast to Iran and Afghanistan. Its name originates from “sumaga,” which means red in Syriac [1].

Among the sumac species, *R. coriaria* L. has the greatest economic importance. In Sicily, this Asian species was first imported by the Arabs; on this island, it grows spontaneously and acquires surprising nutritional characteristics determined by the pedoclimatic conditions [2].

*R. coriaria* is a high shrub or small tree (1–3 m high) with imparipinnate leaves, villose and red fruits with one-seeded drupe, and small greenish-white flowers organized in panicles [3].

Several studies have been carried out in order to identify the major components of the *R.* *coriaria* plant’s different parts [3], its bioactive molecules [4], and its fatty acid composition [5]. However, only few studies have been conducted on Sicilian sumac [6,7], and in particular on its drupes, which are the main endpoints of the proposed study.

Since ancient times, given its nutritional value and its phytochemical components (flavonoids, flavones, anthocyanins, tannins, organic acids, fiber, proteins, volatile oils, nitrites, and nitrates), it has been used both as a spice by mashing and mixing the dehydrated fruits with salt and as a medicinal herb [7,8]. Its ordinary state is a fruit, and, to date, it is economically attractive because of its growing use in several biotechnological applications, from the nutraceutical and food sectors, to cosmetic and pharmaceutical industries as well as in veterinary practices, and in dying leather [3,4,6,9,10].

The *R. coriaria* components, including fatty acids, minerals, fiber, and phytochemicals, are responsible for its several beneficial properties. Its nutritional value makes this plant interesting as a food fortifier or functional food [6]. Its antimicrobial and antioxidant properties make this plant a promising tool as a food preservative [3,11,12]. In addition, its coloring properties and tannins are used in dying and tanning fine leather. The bioactive compounds, which are responsible for antioxidant, antilipidemic, antimicrobial, antiviral, antifungal, and anti-inflammatory activity [11,12,13,14], also make this plant an interesting tool for the pharmaceutical sector.

To date, there is no study investigating the antimicrobial activity of Sicilian sumac, and although several studies have reported on the antimicrobial activity of *R. coriaria* [15,16,17], the proposed study is novel, adding information to the area of interest.

In order to investigate all the promising potential of Sicilian sumac, in the present study, a full characterization of this fruit is reported, including the proximate composition determination, the phenolic and anthocyanin content, and the mineral content. A preliminary comparative antibacterial screening of different sumac extracts was carried out, and its non-toxicity was proven by using the Zebrafish Embryo Toxicity Test (ZFET). Further studies are in progress to have a better understanding of this plant genotype and its possible biotechnological applications.

## 2. Materials and Methods

### 2.1. Reagents and Chemicals

Heptane, methanol, ethanol, and acetone were supplied by PanReac AppliChem (Barcelona, Spain) and J.T. Baker (Phillipsburg, NJ, USA). Ultrapure water (18 mΩ cm resistivity and <5 ppb TOC) was produced by a Barnstead Smart2Pure 12 Water Purification System (Thermo Scientific, Milan, Italy). Reference standards of fatty acids methyl esters (FAMEs, C4–C24) and stock standard solutions of inorganic elements (1000 mg/L in 2% HNO_3_) were obtained from Supelco (Bellefonte, USA). Sugars and polyphenol standards were obtained from Extrasynthese (Genay, France) and Sigma-Aldrich (St. Louis, MO, USA). The Kjeldahl catalyst was supplied by Carlo Erba (Milan, Italy). Methanol, standard gallic acid, cyanidin-3-O-glucoside, and Folin–Ciocalteau reagent were obtained from Sigma-Aldrich (Steinheim, Germany). PTFE syringe filters (0.45 μm) were purchased from Gelman Sciences Inc. (Ann Arbor, MI, USA). High purity water with a resistivity of 10 mΩ cm, nitric acid trace metal analysis grade, and hydrogen peroxide were acquired from J.T. Baker (Milan, Italy). Stock standard solutions of B, Mg, Na, Al, K, Ti, Cr, Mn, Fe, Zn, Ni, As, Sr, Ba, Pb, and Bi (1000 mg/L in 2% nitric acid) were purchased from Fluka (Milan, Italy). The Cd solution (1000 mg/L in 2% nitric acid) and the Hg solution (1000 mg/L in 3% hydrochloric acid) were obtained from Merck (Darmstadt, Germany). MeOH HPLC grade, quercetin, and gallic acid standards were obtained from Sigma-Aldrich (St. Louis, MO, USA).

### 2.2. Plant Material

*R. coriaria* L. drupes (1 kg) were collected in Messina (38°13′31.42″ N 15°32′21.7″ E) in September 2020. The fresh material was immediately dried in the dark at a low temperature and then pulverized.

### 2.3. Proximate Composition Determination

The determination of the proximate composition was carried out according to the AOAC (Association of Official Analytical Chemist) methods [18]. In particular, the following methods were used: dry matter (method 925.10), crude ash (method 923.03), crude protein (method 990.03), crude fiber (method 962.09), starch (method 996.11), and crude fat by Soxhlet extraction (method 960.39 with some modifications). The sumac sample was analyzed in triplicate.

For the fatty acids profiling, 15 g of sample was extracted for 6 h with a Soxhlet apparatus using heptane as solvent. After the extraction, the solvent was eliminated with a rotating evaporator and stored at −18 °C until the chromatographic analysis. The analysis of FAMEs was performed as described by the EU Regulation n. 1833/2015 (European Commission, 2015). One μL of each extract was analyzed by a gas chromatograph (GC) (Dani Master GC1000) equipped with a split/splitless injector and a flame ionization detector (FID) (Dani Instrument, Milan, Italy). A 60 m × 0.25 mm ID 0.20 μm film thickness Supelco SLB-IL100 capillary column (Supelco, Sigma-Aldrich, St. Louis, MO, USA) was used. The chromatographic conditions used were as follows: temperature from 165 °C to 210 °C (10 min held) at 2 °C/min, injector and detector temperature was 250 °C, and helium was at a linear velocity of 30 cm/s. The injection volume was 1 μL with a split ratio of 1:100. The Clarity Chromatography Software v4.0.2 (DataApex, Prague, Czech Republic) was used for data acquisition and processing. The sample was analyzed in triplicate. FAMEs were identified by comparing the retention times of the peaks with those of the standards. The percentage of each FAMEs was calculated by comparison with the corresponding chromatogram peak area. The precision of the method was assessed in terms of RSD% analyzing five times each sample [19].

### 2.4. Total Phenolic and Anthocyanin Content Determination

The total phenolic content of *R. coriaria* L. was determined using the Folin–Ciocalteu method [20]. Briefly, 3 g of dried and minced sample was homogenized with 8 mL of an 80% aqueous methanol solution and placed in a vessel at −20 °C overnight. The sample was then centrifuged (10000 rpm for 15 min), and the supernatant was filtered with a 0.45 µM filter.

The total phenolic content absorbance measurements were registered using a Ultrospec 2100 Pro UV-VIS spectrophotometer (GE Healthcare Ltd., Chicago, IL, USA) at a wavelength of 760 nm. Absorbance values were converted to gallic acid equivalents and expressed as mg/g.

The total anthocyanins content was spectrophotometrically evaluated, as described by Landi et al. [21], in acidified methanol (0.1% HCl, *v*/*v*), and the absorbance was measured at 535 nm. Absorbance values were converted to cyanidin-3-O-glucoside equivalents and expressed as mg/g.

### 2.5. Mineral Element Content Determination

The determination of the mineral elements in the *Rhus coriaria* samples was carried out using an ICP-MS iCAP-Qc spectrometer (Thermo Fisher Scientific, Milan, Italy) equipped with a 27 MHz radiofrequency solid-state generator at 1550 W. A closed vessel microwave digestion system Ethos 1 (Milestone, Bergamo, Italy) was used for the sample digestion. Approximately 0.50 g of *R. coriaria* sample was digested with 7 mL of HNO_3_ (69% *v*/*v*) and 1 mL of H_2_O_2_ (30% *v*/*v*) in a pre-washed PTFE vessel. The sample was then cooled down at room temperature, diluted up to 25 mL with ultrapure water, and filtered with a 0.45 µm filter [19,22]. The certified reference materials were processed using the same conditions. The ICP-MS operating parameters were the incident radiofrequency power 1500 W, plasma gas flow argon (Ar) at 15 L/min, auxiliary gas flow rate (Ar) 0.9 L/min, and carrier gas flow rate (Ar) 1.10 L/min. Helium (He) was the collision cell gas (4 mL/min), and the spray chamber was set at T = 2 °C. The injection volume and sample introduction rate were 200 μL and 1 mL/min, respectively. A full scan mode (dwell time 0.5 s point 1) was used for spectra acquisition. All samples and the analytical blanks were analyzed in triplicate.

Data acquisition was performed using the Qtegra™ Intelligent Scientific Data Solution™ (Thermo Scientific) software. For the quantification, an external calibration procedure was used. The determination of mercury was performed using the automatic mercury analyzer DMA-80 (Milestone Srl, Bergamo, Italy). An aliquot of the sample (100 mg) was placed in a vessel, dried for 3 min at 200 °C, and decomposed at 650 °C for 2 min. The content of Hg was determined by measuring the absorbance at 253.7 nm.

### 2.6. Preparation of Extracts

The dried and ground fruits were extracted with different solvents obtained from Sigma-Aldrich: methanol (SM), ethanol (SE), acetone (SA), methanol and water (SMW), ethanol and water (SEW), acetone and water (SAW), and water (SW). The samples (2 g) in the respective extraction solvents (20 mL) were sonicated for 15 min, filtered with a Whatman filter, dried with a rotary evaporator (BUCHI R-210, Merck KGaA, Darmstadt, Germany), and lyophilized. The resulting dried extracts were used for further microbiological analysis.

### 2.7. Polyphenols Profile in SM

Amounts of 229.16 mg and 224.29 mg of sumac powder were added to 3 mL aliquots of HPLC grade MeOH. The ultrasound-assisted extraction of phenolics from each powder was performed using a Transonic 460 H ultrasonic bath (Elma Hans Schmidbauer, Singen, Germany) at room temperature operating for 15 min at 35 kHz ultrasonic frequency. Then, each sample was centrifuged (5000 rpm, 6 min) using an SL 16 centrifuge (Thermo Fisher Scientific, Waltham, MA, USA). The supernatant was recovered, and to the solid residue, another 3 mL of MeOH were added two times. The methanolic extracts were dried under reduced pressure. Samples were then added with 2 mL of MeOH, filtered, and transferred in a glass vessel. Analyses were carried out using an HPLC Alliance e2695 (Waters, Milford, MA, USA) system equipped with an autosampler, degasser, and column heater coupled with a Q-Tof Premier quadrupole time-of-flight mass spectrometer (Waters, Milford, MA, USA).

The compounds were separated using a 50 × 2.1 mm ID Hypersil GOLD HPLC column (Thermo Fisher Scientific, Waltham, MA, USA) kept at 20 °C. The injection volume was 5 µL. A thermostated auto sampler, kept at 4 °C, was used, and all samples were injected in triplicate. The HPLC eluent was a mix of 0.1% aqueous formic acid solution and 0.1% formic acid in MeOH, with a flow rate of 0.25 mL/min.

Elution started with 95% aqueous formic acid and 5% methanol formic acid, and then isocratic for 1 min. Then, in the following 14 min, the solvent became 100% MeOH, remaining isocratic for the subsequent 5 min (from min 15 to min 20). After 30 s, the eluting solvent mixture was reverted to 95% aqueous formic acid and 5% methanolic formic acid and maintained for another 30 s. Each run lasted 21 min. Every sample was injected three times. The concentration values, which are reported in Table 4, are the arithmetic mean of the values observed in each run.

For the detection of flavonoids and other phenols, the calibration curves of quercetin and gallic acid, both of HPLC purity grade, were used. The calibration curves were performed using a standard solution of quercetin in methanol (1000 ppm 10 mg/10 mL) and a standard solution of gallic acid in methanol (1000 ppm 10 mg/10 mL), respectively. Each calibration curve was obtained using 0.5 ppm, 1 ppm, 5 ppm, 10 ppm, and 20 ppm solutions.

The following compounds were investigated: quercetin, quercetin 2′O-gallate, quercetin glucuronide, quercetin-hexose malic acid, methyl-dihydroquercetin hexoside, kaempferolol, myricetin-rhamnose malic acid, quercetin 3-O-hexuronide, kaempferol 3-O-glucoside, quercetin 3-O-galactoside, myricetin, myricetin 3-O-hexoside, apigenin glucoside, myrecetin O-rhamnosylglucose, phenols, gallic acid, methyl digallate, pentagalloyl-hexoside, p-coumaric acid, peonidin 3-O-hexoside isomer, and vanillic acid.

### 2.8. Antimicrobial Tests

#### 2.8.1. Bacterial Strains

Five *Enterobacteriaceae* isolates, four *Escherichia coli*, and one *Klebsiella pneumonia* were selected to carry out the study. The strains were isolated from poultry in the Regional Veterinary Laboratory of Mostaganem, Algeria, and identified using matrix-assisted laser desorption–ionization time-of-flight mass spectrometry (MALDI-TOF-MS), as previously reported [23]. *E.* *coli* ATCC 25922 and *Staphylococcus aureus* ATCC 6538 (American Type Culture Collection, Rockville, MD, USA) were also tested.

#### 2.8.2. Antimicrobial Susceptibility Testing

Disk diffusion method was used to test and confirm the antimicrobial susceptibility of the Enterobacteriaceae isolates using Muller–Hinton agar (MHA, Oxoid, Milan, Italy) and an incubation time of 16–18 h at 37 °C, following the Clinical and Laboratory Standards Institute Guidelines (CLSI) [24]. The antimicrobial used were: ciprofloxacin (CIP, 5 µg), nalidixic acid (NA, 30 µg), amoxicillin/clavulanic acid (AMC, 20/10 µg), amoxicillin (AML, 25 µg), levofloxacin (LEV, 5 µg), cefotaxime (CTX, 30 µg), sulphonamides (SSS, 300 µg), tetracycline (TE, 30 µg), trimethoprim/sulphamethoxazole (SXT, 1,25/23,75 µg), trimethoprim (TMP, 5 µg), chloramphenicol (C, 30 µg), and neomycin (N, 30 µg) (Bio-Rad, Marnes la Coquette, France). The results were assessed following the CLSI guidelines [24].

#### 2.8.3. Determination of the Antibacterial Activity of all Extracts of *Rhus Coriaria* by Disk Diffusion Assays

The antibacterial activity of the different extracts (SE, SM, SA, SEW, SMW, SAW, and SW) against the selected Enterobacteriaceae isolates was assessed by the disk diffusion method, as previously described [25]. Briefly, the bacterial colonies were suspended in 10 mL of saline water, and the turbidity of the bacterial suspension was adjusted to 0.5 McFarland standard. MHA plates were inoculated with bacteria by spreading overnight cultures on MHA using sterile cotton swabs. Filter paper disks (6 mm diameter; Thermo Fisher, Milan, Italy) containing 10 µL of each extract at a concentration of 10 mg/mL were then applied on the agar plates. Cefotaxime served as a positive control, and a disk impregnated with sterile distilled water was used as a negative control. The plates were incubated for 24 h at 37 °C, and the antibacterial activity was evaluated by measuring the diameters of the inhibition zones. Each assay was performed in triplicate.

#### 2.8.4. Determination of the Minimum Inhibitory Concentration (MIC) of the SM Extract

In order to determine the minimum inhibitory concentration (MIC) of SM, the serial double dilution method was performed according to CLSI guidelines [26]. Briefly, overnight bacterial cultures in log phase were used to prepare the suspension of cells adjusted to 10^6^ CFU in Muller-Hinton Broth (MHB). Serial dilutions were performed in the growth medium in a concentration range between 2000 and 2 μg/mL for the SM extract. Wells containing compound-free MHB with bacteria were used as the positive control. Plates were incubated at 37 °C for 24 h. The MIC value was defined as the lowest concentration of the tested compound that inhibits the growth of bacteria at the end of the 24 h incubation. MICs were determined in triplicate. The MIC was defined as the lowest concentration inhibiting the visible growth of the tested strains after incubation.

### 2.9. Embryo Acute Toxicity Test

The embryo acute toxicity test was carried out according to the Organisation for Economic Cooperation and Development (OECD) guidelines for the testing of chemicals [27]. The ZFET was conducted on fertilized eggs from the Centre for Experimental Fish Pathology of Sicily (CISS, Sicily, Italy). Adult zebrafish (*Danio rerio*) were kept in a standalone facility (ZebTec, Tecniplast, West Chester, PA, United States) in water-controlled conditions: temperature 28 °C, conductivity 600 µS/cm, pH 7.5, and 14/10 h dark/light regimen. Twice a day, fish were fed with *Artemia salina* at 3% of body weight and Gemma micro 300 (Skretting, Varese, Italy). Following mating, the eggs were placed in steel grids inside tanks to avoid predation by adults and to guarantee their collection. The fertilized eggs were collected using a stereomicroscope (Leica M205 C) and exposed to *R. coriaria* extract, which was previously prepared at a concentration of 9.37 µg/mL, in a sterilized embryo medium (15 mM NaCl, 0.5 mM KCl, 1 mM CaCl_2_, 1 mM MgSO_4_, 0.15 mM KH_2_PO_4_, 0.05 mM Na_2_HPO_4_, 0.7 mM NaHCO_3_; pH 7.3). The control group was held in an embryo medium. The Fish Embryo Acute Toxicity (FET) was performed, as described by Pecoraro et al. [28]. Right after the fertilization, embryos were collected, bleached as reported by Westerfield [29], and distributed as one embryo per well into 24-well plates (LABSOLUTE, Th. Geyer GmbH & Co.KG, Berlin, Germany). Embryos were incubated with a 10/14 h dark/light regimen at 26 °C for 96-hours post-fertilization (hpf). The test solutions and controls were replaced daily [27]. The exposure period started from 180 min post-fertilization and ended at 96 h. The following endpoints were used to evaluate the toxicity: embryo coagulation, tail non-detachment, somite formation lack, heartbeat non-detection, and the hatched embryos number. Acute toxicity was determined at the end of the exposure period.

### 2.10. Statistical Analysis

The existence of significant differences in the antimicrobial activity of the extracts was assessed by one-way ANOVA using SIGMAPLOT, version 14.0 (Systat Software Inc., San Jose, California, USA). Tukey’s HSD was used as a post hoc test. The level of significance was set at *p* ≤ 0.05.

## 3. Results and Discussion

### 3.1. Chemical Characterization

The proximate composition and fatty acid profile for *Rhus coriaria* from Sicily are reported in Table 1 and Table 2, respectively. 

These results show that sumac represents a source of dietary fiber, which may be useful for relieving gastrointestinal upset [30].

In line with previous studies, fats represent the second most abundant compounds [5,9]. The fatty acid composition is reported in Table 2. As it can be observed, sumac fruits contain 65.09 ± 1.67% of polyunsaturated fatty acids, and the contents of linoleic (omega 6) and α-linolenic acid (omega 3) are 30.82 ± 1.21% and 1.85 ± 0.07%, respectively. This result confirms that this fruit could represent a source of unsaturated fatty acids, as previously reported [9]. Furthermore, oleic acid is the most abundant fatty acid, representing 31.67 ± 1.29%.

Total phenolic and total anthocyanin contents of *R. coriaria* extract are 71.69 ± 1.23 mg/g as gallic acid equivalent, and 6.71 ± 0.12 mg/g as cyanidin-3-O-glucoside equivalents, respectively. These two classes of compounds in sumac are the principal constituents responsible for its phytochemical activity. *R. coriaria* is, hence, a source of healthy substances and is useful in various fields and applications.

In agreement with a previous study [31], our results confirm that sumac is a good source of minerals, so it could be a suitable tool to increase dietary mineral intake. As reported in Table 3*, R. coriaria* is mainly rich in potassium, calcium, magnesium, and phosphorus, followed by aluminum, iron, sodium, boron, and zinc.

The polyphenolic composition of methanolic sumac extract is reported in Table 4. The total polyphenols account for 71.69 mg/g. The flavonoids and phenols represent 18.48% and 81.52%, respectively. The first component identified was gallic acid, followed by quercetin, methyl digallate, pentagalloyl-hexoside, and kaempferol 3-O-glucoside.

### 3.2. Antimicrobial Activity

In order to analyze the antimicrobial activity of sumac, a comparative study was conducted by evaluating the antibacterial potential of six different extracts against foodborne pathogens.

The isolates involved one *S. aureus* isolate, one *K. pneumoniae* isolate, and five *E. coli* isolates.

The antimicrobial susceptibility test showed that the Enterobacteriaceae isolates from poultry (*E coli* and *K. pneumoniae* strains) were resistant to AML and N. The Enterobacteriaceae isolates tested were found to be resistant to three or more antimicrobial agents that belong to dissimilar antibiotic classes and are multidrug resistant (MDR). The antimicrobial profile of the tested microorganisms is reported in Table 5.

These results, showing the multi-resistance of the majority of the isolates, confirm previous studies that reported a high level of multidrug resistant Enterobacteriaceae isolates from poultry in Algeria [23,32]. The causes of this antibiotic resistance phenomenon are very different, and the important cause is the massive and inappropriate use of antibiotics. Currently, antimicrobial resistance represents a growing global concern, and the development of effective therapeutic options against MDR bacteria is a public health priority.

Plant extracts can be valuable alternatives to antibiotics [33,34]. Recently, sumac has gained more attention due to its high amount of polyphenols. Numerous studies have been conducted to study the in vitro antimicrobial effectiveness of *Rhus coriaria* extracts against several bacterial species [14,35,36], while limited data are available on their effect against MDR bacteria. In this study, the antibacterial activity of *Rhus coriaria* extracts was tested against five MDR Enterobacteriaceae isolates, *S. aureus* ATCC 6538, and *E. coli* ATCC 25922. The results of the antibacterial activity of *Rhus coriaria* extracts are summarized in Table 6, which indicates that almost all the extracts exerted antibacterial activity against all the tested strains.

The higher antimicrobial activity was obtained by using SM and SE. In particular, SE presents growth inhibition zones ranging from 14 to 16 mm and SM ranging from 22 to 25 mm. Given the higher antimicrobial activity of the SM, its MIC was evaluated, and the results are reported in Table 7. These results are in line with a study on Syrian sumac antimicrobial activity, which reports that the antimicrobial activity of the methanol extract was the most effective [37]. In addition, further studies reported on the higher amount of phenolics and flavonoids contained in the methanolic extract compared with the ethanolic one [37,38]. Several studies have described the antibacterial activity of the fractions of sumac extract, highlighting how some compounds, due to their polarity, can act only on Gram positive bacteria, while some others, such as gallic acid, can affect both Gram positive and Gram negative bacteria; nevertheless, their antibacterial activity is not so strong [38].

Several studies have also reported on the efficacy of the total extract and attribute its biological activity to its content in phenolics, which are the major extract fraction [39,40,41].

The maximum inhibitory action was observed at a concentration of 9.37 μg/mL for all strains except for *E coli* (S2/15), which presented an MIC of 4.68 μg/mL. This finding supports the use of *Rhus coriaria* in traditional medicine as a bactericide agent. A study reported that the water extract of *R. coriaria* had an effective in vitro antibacterial power against *S. aureus*, *P. aeruginosa*, and *S. aureus* (MRSA) [35]. Another study reported that the extract showed strong antibacterial activity against Gram positive and Gram negative bacteria, with MIC < 0.78% [42]. A similar study assessed sumac’s methanolic extract antibacterial power as having the highest inhibitory activity. In all sumac extracts, increasing the concentration of sumac causes an increase in antibacterial power [14]. In addition, the methanolic extract of sumac leaves revealed antibacterial activity against *E. coli* and *S. aureus.* A MIC of 312 μg/mL was reported, although the inhibitory effect was only bacteriostatic, and the bactericidal effect was observed at a concentration of 2500 μg/mL [36].

The results of our study, highlighting the higher activity of the methanol extract, are therefore in line with previous comparative studies that showed that methanolic extracts of sumac contain a higher content of flavonoids and phenolics when compared with other extracts [13,14].

### 3.3. Fish Embryo Acute Toxicity (FET)

The evaluation of the toxicological profile of medicinal plant extract is of utmost importance. Zebrafish (*Danio rerio*) is one of the main study models [43]. Given that the embryo develops quickly outside the mother and that this is visually evident, it is certainly usable for testing and observation. Being that the ZFET is a valid alternative method to animal tests [44,45], the non-toxicity of the *R. coriaria* genotype from Sicily was assessed and confirmed by analyzing the effect of the extract on zebrafish larvae. To date, there is no evidence of the toxicity of Sicilian sumac.

**Figure 1:**
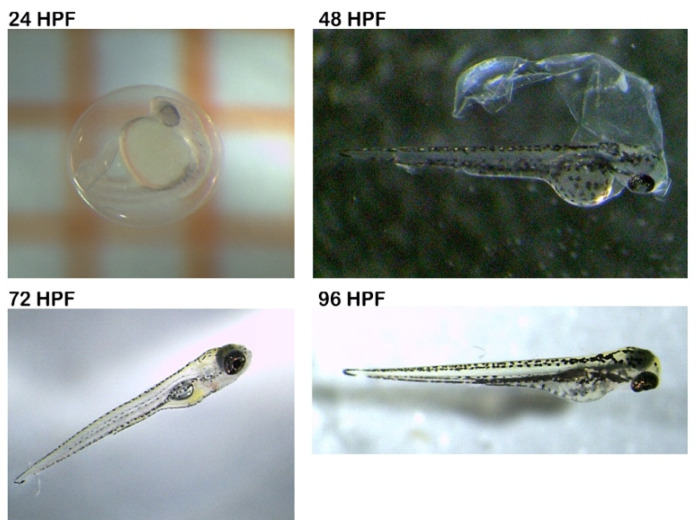
Development of embryos. Hpf: hours post-fertilization.

In this study, fertilized zebrafish embryos were exposed to SM extract from *R. coriaria* at a concentration of 9.37 µg/mL. According to our results, the extract was found to be non-toxic using zebrafish FET assay. During the 96 h of exposure, no visible toxic effects of this extract on the development of embryos were observed (Figure 1). The mortality was 5% (one larva) for the whole test period. During the observation under the stereomicroscope, it was observed that at 48 hpf (hours post-fertilization), the hatched larvae were 95%.

According to the OECD guidelines, the *R. coriaria* extract obtained from the Sicilian genotype did not induce any toxic effect on zebrafish embryos and larval development. These results are in line with other findings reporting the safe and even beneficial effects of the *R. coriaria* extract on both humans and animals [5]. Other studies evidenced no acute toxicity of the extract in rat model experiments and, in addition, showed beneficial cardioprotective and hepatoprotective properties under hypercholesterolemic conditions [46]. Another study using a diabetic rats model and testing 250, 500, and 1000 mg/kg of the plant extract reported good tolerance and a non-lethal oral uptake of this extract, even at 1000 mg/kg, showing not only no signs of toxicity and mortality after 3 days of daily extract administration but also a positive effect on diabetes and diabetes-related complications [47]. Taken together, these results suggest the safety of this plant.

## 4. Conclusions

This study provides a characterization of Sicilian sumac drupes, including the proximate composition determination, the phenolic and anthocyanin content, and the mineral content, and suggests its potential use in the food supplement area [48,49,50,51]. In addition, it offers for the first time a preliminary screening of its antimicrobial activity against MDR Enterobacteriaceae and proves the non-toxicity of this food matrix. Taken together, the results show the potential of sumac as a functional food supplement and the application of the sumac extract in the food industry, not only as a food additive but also as an efficient and natural food preservative.

## Figures and Tables

**Table 1:** Proximate composition of *Rhus coriaria* from Sicily.

g/100 g	Sumac Fruits
Crude fiber	33.21 ±1.02
Ash	4.78 ±0.29
Crude oil	9.56 ± 0.72
Moisture	6.64 ± 0.03
Crude protein	3.47 ± 0.19
Carbohydrates	ND
Crude energy (Kcal)	ND

ND, not determined.

**Table 2:** Fatty acid profile of *Rhus coriaria* from Sicily.

Fatty Acid g/100 g	Sumac Fruits
Miristic acid	0.38 ± 0.08
Palmitic acid	31.25 ± 0.47
Palmitoleic acid	0.75 ± 0.15
Stearic acid	3.28 ± 0.55
Oleic acid	31.67 ± 1.29
Linoleic acid	30.82 ± 1.21
Linolenic acid	1.85 ± 0.07
∑ TUFA	65.09 ± 1.67
∑ TSFA	34.91 ± 1.04

**Table 3:** Mineral elements (mg/kg) in *Rhus coriaria* fruits powder.

Cr	B	Na	Mg	Al	K	Ti
0.040 ± 0.00	0.770 ± 0.09	3.980 ± 0.11	41.870 ± 3.55	4.010 ± 0.24	266.91 ± 15.55	0.480± 0.56
Mn	Fe	Ni	Zn	As	Sr	Cd
0.410 ± 0.06	2.950 ± 0.13	0.020 ± 0.00	0.360± 0.06	0.001 ± 0.00	5.390 ± 2.49	0.003± 0.00
Ba	Pb	Bi	Hg	Li	V	Co
0.270 ± 0.08	0.010 ± 0.00	0.004± 0.00	0.008± 0.00	0.010 ± 0.00	0.010 ± 0.00	0.001 ± 0.00
Cu	Se	Mo	Sb	Tl	P	Ca
0.120 ± 0.00	0.065 ± 0.00	0.005 ± 0.00	0.001 ± 0.00	0.002 ± 0.00	39.70 ± 3.05	215.53 ± 16.78

**Table 4:** Polyphenolic compounds in *Rhus coriaria* SM extract.

Compounds	SM Extract (mg/g)
Flavonoids	
Quercetin	23.13 ± 0.02
Quercetin 2′O-gallate	5.30 ± 0.02
Quercetin glucuronide	1.71 ± 0.01
Quercetin-hexose malic acid	11.11 ± 0.01
Methyl-dihydroquercetin hexoside	18.34 ± 0.02
Kaempferolo	3.34 ± 0.01
Myricetin-rhamnose malic acid	11.58 ± 0.01
Quercetin 3-O-hexuronide	2.84 ± 0.02
Kaempferol 3-O-glucoside	99.86 ± 0.01
Quercetin 3-O-galactoside	160.53 ± 0.02
Myricetin	2.71 ± 0.02
Myricetin 3-O-hexoside	18.55 ± 0.01
Apigenin glucoside	20.86 ± 0.01
Myrecetin O-rhamnosylglucose	4.34 ± 0.02
Phenols	
Gallic acid	142.549 ± 0.02
Methyl digallate	110.96 ± 0.01
Pentagalloyl-hexoside	128.09 ± 0.01
p-Coumaric acid	10.48 ± 0.01
Peonidin 3-O-hexoside isomer	4.54 ± 0.02
Vanillic acid	5.76 ± 0.02

**Table 5:** Antimicrobial resistance profile.

Strains	Antimicrobial Resistance Profile
*Escherichia. coli* (S12/15)	NA, CIP, AML, AUG, SXT, TE, N
*Escherichia. coli* (S34/16)	NA, CIP, N
*Escherichiacoli* (S6/15)	NA, CIP, AML, AUG, TE, N
*Escherichia. coli* (S2/15)	NA, CIP, AML, AUG, SXT, TE, N
*Klebsiella pneumoniae*	AML, SXT, TE, C, N

NA, nalidixic acid; CIP, ciprofloxacin; AML, amoxicillin; AUG, amoxicillin-clavulanic acid; SXT, trimethoprim-sulfamethoxazole; TE, tetracycline; N, neomycin; C, chloramphenicol.

**Table 6:** Antibacterial activity of extracts from *Rhus coriaria* evaluated by disk diffusion assay.

Strains	Radius of Inhibition in mm (Mean of Three Tests)						
	SA	SE	SM	SAW	SEW	SMW	SW
*Staphylococcus aureus* ATCC 6538	10 ^A^^,B,C,D,H,P^	11 ^D^^,E,F,G,I,R^	19 ^H^^,I,L,N,O^	9 ^A^^,E,L,N,Q^	10 ^B^^,F,M,S^	17 ^O^^,P,Q,R,S^	5 ^C^^,M^
*Escherichiacoli* ATCC 25922	13 ^A,B,C^	12 ^A, D, E,G^	15	11 ^D,F,H^	11 ^B,^^E,F,I^	12 ^C,G,H,I^	4
*Klebsiella pneumoniae*	9 ^A^^,B^	14	22	8 ^A, C^	11	19	8 ^B^^,^^C^
*Escherichiacoli* (S12/15)	15 ^A^	19 ^B^	25 ^B^	14	17 ^A^	22	7
*Escherichiacoli* (S34/16)	16 ^A^^,B^	17 ^B^	24	8	14 ^A^	21	5
*Escherichiacoli* (S6/15)	14 ^A^	20 ^B^	24	12 ^B^	15 ^A^	20	7
*Escherichiacoli* (S2/15)	12 ^A^^,B^	23	22	11 ^A^	16 ^B^	19	6

SA, acetone extract; SE, ethanol extract; SM, methanol extract; SAW, acetone + water extract; SEW, ethanol + water extract; SMW, methanol + water extract; SW, water extract. Means sharing the same capital letters in the raw are not significant at *p* < 0.05 according to Tukey’s HSD test.

**Table 7:** Antibacterial activity of SM from *Rhus coriaria* evaluated by serial double dilution method.

Strains	MIC (μg/mL)
*Staphylococcus aureus ATCC 6538*	9.37
*Escherichiacoli* ATCC 25992	9.37
*Klebsiella pneumoniae*	9.37
*Escherichiacoli* (S12/15)	9.37
*Escherichiacoli* (S34/16)	9.37
*Escherichiacoli* (S6/15)	9.37
*Escherichiacoli* (S2/15)	4.68
